# INTERGENERATIONAL EQUITY: AN URGENT CALL FOR AGING POLICIES IN SUB-SAHARAN AFRICA

**DOI:** 10.1093/geroni/igae098.3178

**Published:** 2024-12-31

**Authors:** Abraham Teshome, Dolapo Adeniji, Gifty Ashirifi, Margaret Adamek

**Affiliations:** Indiana University Indianapolis, Indianapolis, Indiana, United States; Indiana State University, Avon, Indiana, United States; Indiana University Indianapolis, Indianapolis, Indiana, United States; Indiana University Indianapolis, Indianapolis, Indiana, United States

## Abstract

Contrary to popular belief, family-based care and support for older people in Sub-Saharan Africa is gradually eroding due to various factors, including urbanization, migration, and modernization. As the issues of older people and aging are not mainstreamed into national economic and development agenda and policies, most older people in the region live in abject poverty, which has undermined their healthy aging and quality of life. Although many Sub-Saharan African countries are signatories to the 2002 Madrid International Plan of Action on Ageing (MIPAA), only a few have developed national aging policies to meet the needs of their older population. Against this backdrop, we conducted cross-sectional qualitative study to learn why public policy in Sub-Saharan Africa is sluggish in responding to the needs of the region’s growing older population. We conducted an online survey (n=78) and three FGDs (n=13) with African scholars and practitioners from 12 nations. Based on descriptive and thematic analyses, our study revealed five themes: a) lack of political will, b) disconnect between researchers and policymakers, c) lack of research and comprehensive data related to older people, d) inappropriate aging policy, and e) budget constraints. Our study’s findings call for more research and comprehensive data regarding the needs and challenges of the region’s older population, policy advocacy, and awareness-raising about older adults’ issues among policymakers, involvement of all stakeholders in aging policy development, and regular dialogues between policymakers, researchers, older adults, and other stakeholders.

